# Systematic detection of putative tumor suppressor genes through the combined use of exome and transcriptome sequencing

**DOI:** 10.1186/gb-2010-11-11-r114

**Published:** 2010-11-25

**Authors:** Qi Zhao, Ewen F Kirkness, Otavia L Caballero, Pedro A Galante, Raphael B Parmigiani, Lee Edsall, Samantha Kuan, Zhen Ye, Samuel Levy, Ana Tereza R Vasconcelos, Bing Ren, Sandro J de Souza, Anamaria A Camargo, Andrew JG Simpson, Robert L Strausberg

**Affiliations:** 1 Ludwig Collaborative Group, Department of Neurosurgery, Johns Hopkins University, 1550 Orleans Street, Baltimore, MD 21231, USA; 2 J. Craig Venter Institute, 9704 Medical Center Drive, Rockville, MD 20850, USA; 3 Ludwig Institute for Cancer Research, São Paulo Branch at Hospital Alemão Oswaldo Cruz, Rua João Julião 245, 01323-903 São Paulo, Brazil; 4 Ludwig Institute for Cancer Research, San Diego Branch, 9500 Gilman Drive, La Jolla, CA 92093-0660, USA; 5 Scripps Translational Science Institute, 3344 North Torrey Pines Court, La Jolla, CA 92037, USA; 6 Laboratório Nacional de Computação Científica, Laboratório de Bioinformática, Av. Getúlio Vargas 333, Petrópolis, RJ 25651-075, Brazil

## Abstract

**Background:**

To identify potential tumor suppressor genes, genome-wide data from exome and transcriptome sequencing were combined to search for genes with loss of heterozygosity and allele-specific expression. The analysis was conducted on the breast cancer cell line HCC1954, and a lymphoblast cell line from the same individual, HCC1954BL.

**Results:**

By comparing exome sequences from the two cell lines, we identified loss of heterozygosity events at 403 genes in HCC1954 and at one gene in HCC1954BL. The combination of exome and transcriptome sequence data also revealed 86 and 50 genes with allele specific expression events in HCC1954 and HCC1954BL, which comprise 5.4% and 2.6% of genes surveyed, respectively. Many of these genes identified by loss of heterozygosity and allele-specific expression are known or putative tumor suppressor genes, such as *BRCA1*, *MSH3 *and *SETX*, which participate in DNA repair pathways.

**Conclusions:**

Our results demonstrate that the combined application of high throughput sequencing to exome and allele-specific transcriptome analysis can reveal genes with known tumor suppressor characteristics, and a shortlist of novel candidates for the study of tumor suppressor activities.

## Background

Cancer arises from the accumulation of genetic and epigenetic changes that disrupt the normal regulatory controls in cells. Recently, next generation sequencing technology has been employed to identify variations in protein-coding sequences and genome structure for several types of cancers [1-9]. These studies have revealed the effectiveness of high throughput sequence analysis to identify somatic genomic alterations, such as point mutations, and structural variations, including gain and loss of chromosome regions. An important finding is that integrated analysis of the various somatic alterations is key for identifying genes that may drive cancer development and progression through oncogenic or tumor suppressor functions. Here, we combine the detection of two types of molecular events, loss of heterozygosity (LOH) and allele-specific expression (ASE), to identify genes with known and potential tumor suppressor characteristics.

The common feature of LOH and ASE is loss of expression from one allele, which has frequently been observed for tumor suppressor genes. In ASE, a dominant gene product is expressed from the selected allele. For some genes, subtle changes in expression level and balance between alleles could be physiologically significant. Haploinsufficiency of many tumor suppressor genes promotes tumorigenesis and metastasis [10].

ASE is classically associated with epigenomic regulation, and can be heritable. Two extreme examples are inactivation of genes on the X chromosome in female cells, and imprinting of autosomal genes [11]. ASE can arise from epigenetic modification of the genome, including DNA methylation and histone modification [12,13]. Genetic variations in the coding or non-coding regions of a gene are likely to influence these epigenetic controls [14]. However, allelic differences in gene expression are variable among populations and among tissue types [15,16], suggesting that ASE can be context specific with regard to cell type, cell differentiation status, and exposure to external stimuli. Recently, subtle differences in allelic expression have been detected for numerous human genes, and in a few cases, have been associated with a genetic predisposition to disease, including cancer [17,18].

Previously, genome-wide quantification of ASE events has been estimated by hybridization-based [15,19,20] and sequencing-based [17] methodologies. Recently, several studies have highlighted specific roles of ASE in oncogenesis, many as germline ASE [18,21,22]. Here, we have applied comprehensive sequence-based approaches using exome capture and transcriptome sequencing in a breast cancer cell line, HCC1954, to identify potential cancer-specific and somatically driven LOH and ASE events, and to discern their functional characteristics. This cell line, derived from a ductal breast carcinoma, is estrogen negative, progesterone receptor negative and ERBB2 positive, and has been particularly well studied at the molecular level [2,7,23]. A matching control cell line, HCC1954BL, which was established from lymphoblast cells of the same patient, was studied in parallel. We demonstrate that combined analysis of exome and transcriptome sequences provides a dynamic image of tumor cells that is particularly relevant to tumor suppressor networks.

## Results

### Application of exome sequencing to LOH detection

For both HCC1954 and HCC1954BL, exome capture was performed with the NimbleGen 2.1 M array, followed by 454 Titanium sequencing of captured DNA from each cell line. The 454 reads were mapped uniquely to the human reference genome (hg18) using GS Reference Mapper (gsMapper). Variants and variant allele frequencies were called from high-confidence single nucleotide variants (SNVs) detected by gsMapper (see Materials and methods; Tables S1 and S2 in Additional file 1) and were used for the subsequent analysis. Table 1 summarizes the sequencing and mapping results from the exome sequencing effort. We identified 13,102 and 14,219 SNVs in the 26.4 Mb of primary target sequence for HCC1954 and HCC1954BL, respectively.

**Table 1:** Statistics of exome sequencing and reads mapping

	HCC1954	HCC1954BL
Number of 454 reads	6,878,120	6,658,357
Total bases pairs	2,588,213,873	2,325,966,906
Uniquely mapped reads	6,645,304 (97%)	6,385,651 (96%)
Reads uniquely mapped to primary targets	4,806,828 (70%)	4,310,274 (65%)
Target coverage	94.8%	96.0%
Mean target coverage	19.4×	18.1×
Median target coverage	16×	16×
Coverage enrichment by exome sequencing	23×	24×
Total high-confidence (HC) SNVs (known SNVs)	13,102 (12,145)	14,219 (13,309)
HC heterozygous SNVs (known SNVs)	5,602 (4,954)	8,203 (7,408)
HC heterozygous SNVs in CDS (known SNVs)	5,329 (4,709)	7,848 (7,082)

CDS, coding sequence; SNV, single nucleotide variant.

**Figure 1:**
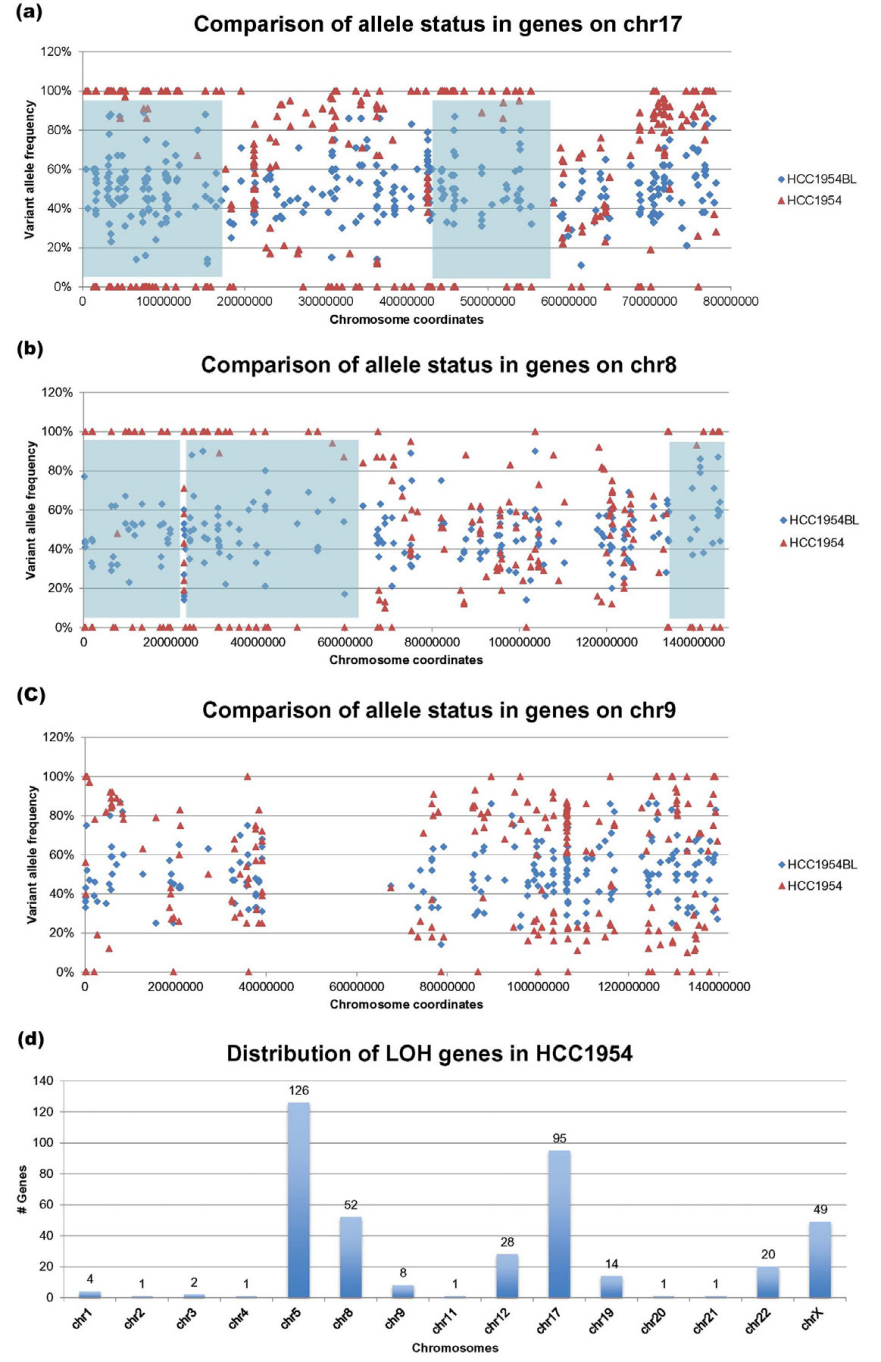
**Exome-based loss of heterozygosity events detected in HCC1954**. **(a-c) **Comparison between variant allele frequencies of the same locus between HCC1954 and HCC1954BL for chromosome 17 (a), chromosome 8 (b) and chromosome 9 (c). Blue shaded areas represent large LOH regions. **(d) **Distribution of LOH genes across the HCC1954 chromosomes.

With variant allele frequencies ranging from 10% to 90%, 8,754 preliminary heterozygous SNVs were defined in HCC1954BL. For these mostly exomic SNVs in HCC1954BL, we examined the variant allele frequencies at the corresponding loci in HCC1954, requiring ten unique reads of the same genotype to support a homozygous locus (*P *< 0.001). Comparison of variant allele frequencies between HCC1954 and HCC1954BL identified many LOH events in large genomic clusters across the genome and in isolated genes (Figure 1; Figure S1 in Additional file 2). LOH occurred on all chromosomes, with particularly large blocks on chromosomes 5, 8, 12 and 17. Our results are in agreement with LOH data generated using the Affymetrix SNV array 6.0 [24] for regions of large genomic deletions on chromosomes 5, 8, 12, 17, 19, 22 and X by approximate genome coordinates. However, there are discrepancies for chromosome 9. Our data do not support a major LOH block on 9q (Figure 1c). As expected, 9q12 and 9q13 are gene deserts. From 9q21 to the telomeric end of 9q, allele variations are consistently detected across this region.

To identify specific genes displaying LOH in HCC1954, we used more stringent criteria that required a heterozygous locus with variant allele frequency between 20% and 80% in HCC1954BL, together with homozygosity in HCC1954 (*P *< 0.001). In HCC1954BL, 8,203 heterozygous SNV loci were defined, with 7,848 in the coding sequence (CDS). LOH events are thus detected in 403 genes as revealed by 609 SNVs, among which 544 are known SNPs (Tables S1, S2 and S3 in Additional file 1). Most of the LOH genes are clustered together in large blocks as described above. For those single LOH genes that are isolated, we also required that the homozygous SNV in HCC1954 has been defined previously in dbSNP, that no conflicting allelic status is detected within 25 kb, and that the homozygosity of the SNV locus is supported by transcriptome reads. Genes with LOH are located on 15 chromosomes, with most on chromosomes 5 and 17, including *BRCA1 *(Figure 1a; Additional file 2). Using the same criteria, only one LOH gene was detected in HCC1954BL (*RRAS2*).

We compared the allelic status of SNPs that were defined in our LOH analysis with those that were genotyped by Affymetrix Genome-Wide Human SNP Array 6.0 [GEO:GSE13373]. In HCC1954BL, heterozygous SNP calls matched perfectly between the two platforms for all 345 known SNPs that were shared. Only one of 224 homozygous SNPs identified by SNP array was revealed as heterozygous by sequencing. For HCC1954, heterozygous SNPs calls were also 100% consistent between the two platforms for all 172 SNPs that are shared. However, 29 of 270 (11%) homozygous SNPs, defined by SNP array, were identified as heterozygous by exome sequencing. Thus, there was a high level of consistency between the two platforms, with sequencing possibly providing greater sensitivity for cancer genomes that carry a wide spectrum of copy number variations.

Out of the 403 LOH genes in HCC1954, 267 have expression in the transcriptome with at least 1× average base pair coverage per gene. To systematically assess the putative biological functions of the LOH genes, we performed Gene Ontology and pathway (KEGG) analysis on the 403 LOH genes. A selection of representative molecular functions is presented in Table 2. The top category of functional network is molecular transport and drug metabolism with 29 LOH genes. Thirteen LOH genes, including *BRCA1 *and *MSH3*, are in the DNA replication, recombination and repair pathway.

**Table 2:** Top categories of general molecular types

General molecular function	Number of genes	Examples
LOH genes		
Enzyme	80	*USP26*, *INPP5K*, *PTPRS*, *MAT2B*
Kinase	25	*CDK7*, *DGKE*, *MAP2K4*, *PDGFRL*
Transporter	21	*ATP2B3*, *SLC36A3*, *SIL1*, *ABCA7*
Transcription regulator	20	*BRCA1*, *FOXD4*, *SOX5*, *VEZF1*
G-protein coupled receptor	19	*GPR174*, *OR1A2*, *TAS2R7*, *GRM6*
Transmembrane receptor	9	*SEMA5A*, *IL31RA*, *ITGB3*, *OSMR*
Cytokine	7	*IL3*, *ERBB2IP*, *EDA*, *CXCL16*
Ion channel	6	*CCT8L2*, *CNGA2*, *GABRA6*, *GRIN3B*
		
ASE genes		
Enzyme	18	*MGMT*, *PLCH1*, *GUCY1A3*, *PYGL*
Transcription regulator	7	*CTBP2*, *SMARCA4*, *BCLAF1*, *SPEN*
Kinase	6	*FGFR2*, *FGFR4*, *IP6K2*, *TAOK1*
Transporter	5	*SLC44A5*, *SLC25A5*, *SNX15*, *LBP*
Transmembrane receptor	3	*HLA-DQA1*, *HLA-A*, *TNFRSF10D*
G-protein coupled receptor	2	*ADORA1*, *GPR107*

Genes are binned exclusively into each category based on their primary molecular function terms. LOH, loss of heterozygosity; ASE, allele-specific expression.

### mRNA allelotyping by transcriptome sequencing

High throughput sequencing of transcriptomes for HCC1954 and HCC1954BL was performed, with 14.0 Gbp and 13.6 Gbp generated by short read paired-end sequencing, respectively. Sequence reads were subsequently aligned to the RefSeq gene set [25] as well as to the human reference genome with CLCBio Genomic Workbench (see Materials and methods). With a cutoff of 1× average coverage across each gene, 14,397 and 14,251 genes were found to be expressed in HCC1954 and HCC1954BL, respectively. These numbers are comparable to previous transcriptome studies [26,27]. The average base pair coverage for the detected transcriptome is approximately 120× for HCC1954 and 115× for HCC1954BL. For HCC1954, 7,173 transcripts displayed SNVs at a minimum of one locus per transcript, indicating that these genes are expressed from both alleles (see Materials and methods). The remaining 7,224 transcripts lack detectable allelic variation. These include many cases in which coverage is not sufficient to make a call for allelic variation. For HCC1954BL, 7,595 genes have detectable allelic variation within transcribed regions, while variants were not detectable in transcripts of 6,656 genes.

### Allele-specific expression detection

**Figure 2:**
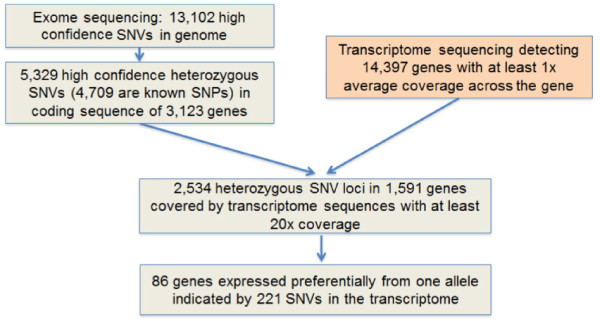
**Schematic diagram of allele-specific expression events detected by combination of exome sequencing and transcriptome sequencing**.

**Figure 3:**
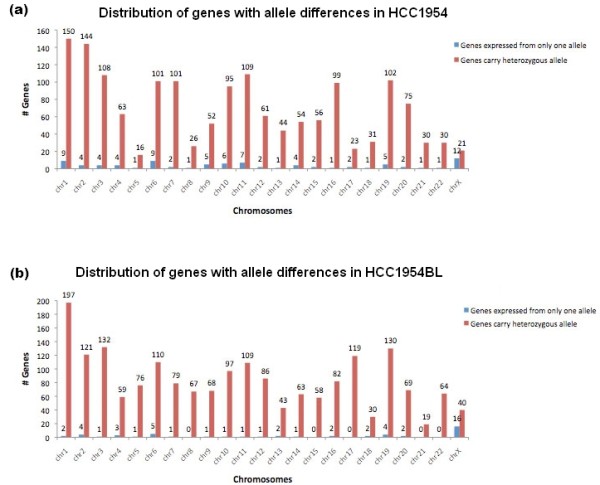
**Distribution of genes carrying high-confidence heterozygous alleles and genes under allele-specific expression**. **(a) **In HCC1954; **(b) **in HCC1954BL.

With genotyping information acquired by exome sequencing, the ASE mining process is summarized in Figure 2 for HCC1954. We started with 3,123 genes that carry heterozygous loci at the genomic level as shown by 5,329 SNVs detected in the CDS by exome sequencing. Of 5,329 SNVs, 620 (11.6%) have not been reported in dbSNP130 [28]. The 5,329 SNVs were checked for coverage by transcriptome sequence reads. The binomial test was utilized to calculate the distribution of alleles represented by numbers of reads that are expected by chance, and led to the requirement that each SNV locus was covered by at least 20 transcriptome reads. Of 5,329 SNVs, 2,534 SNVs in 1,591 genes met this minimum coverage requirement. A stringent criterion of allele drift ratio (< 0.2 or >0.8) was applied to all expressed variant alleles to be considered as biased. A binomial test was then calculated with two adjustments to determine if there was biased expression from one allele (see Materials and methods). Due to the pseudo-tetraploid nature of the HCC1954 genome and copy number changes across the genome, the probability of success (p_s) ratio was adjusted based on variant allele frequency from the exome sequencing data instead of the static 0.5 for the normal diploid genome. A second adjustment was made to correct for multiple sampling. With a cutoff of *P *< 0.05, 221 SNVs in 86 genes were found to be expressed preferentially from one allele (Table S5 in Additional file 1). Table 3 lists a selection of ASE genes with the most significant *P*-values (*P *< 0.001) in HCC1954. Consistently, all the ASE calls were supported by the transcriptome sequence across the entire transcript length, including SNVs detected by the transcriptome reads in the 5' and 3' UTRs for the 86 genes. Out of 221 SNVs utilized in the ASE analysis, 72 (33%) are novel. The higher ratio of novel SNVs seen in ASE genes compared to that of 11% in the whole exome analysis can be explained by the fact that, among 86 ASE genes reported, 13 ASE genes carry multiple novel SNVs. This led to a large random standard deviation. The chromosomal distributions of the 86 ASE genes, and the 1,591 transcripts containing >20× coverage of CDS SNVs is shown in Figure 3a.

**Table 3:** Selected list of allele-specific expression genes detected in the HCC1954 cell line

Gene	Number of reads ratio major/minor	**Chr**.	Gene product	Non-ASE *P*-value	Known SNV ID
***CTBP2***	404/0	10	C-terminal binding protein 2	0.000	3 novel SNVs
	415/1				
	395/0				
***HLA-A***	267/4	6	Major histocompatibility complex, class I, A	0.000	rs2231114
	546/0			0.000	rs1059517
***TAOK1***	223/16	17	TAO kinase 1	0.000	rs508706
***PLAU***	140/0	10	Plasminogen activator, urokinase	0.000	rs2227568
***PODXL2***	124/1	3	Podocalyxin-like 2	0.000	rs920232
***ITGB2***	53/2	21	Integrin, beta 2	0.000	rs11088969
***LXN***	45/1	3	Latexin	0.000	rs8455
***SNUPN***	48/2	15	Snurportin 1	0.000	rs11547316
***RAB3A***	50/5	19	RAB3A, member RAS oncogene family	0.000	rs1046565
***KIN***	31/0	10	KIN, antigenic determinant of recA protein homolog (mouse)	0.000	rs61752337
***GLB1L2***	26/0	11	Galactosidase, beta 1-like 2	0.000	rs3741097
***MGMT***	21/0	10	O-6-methylguanine-DNA methyltransferase	0.001	rs2308327
*PLEKHA6*	340/1	1	Pleckstrin homology domain containing, family A member 6	0.000	rs33911350
*THNSL2*	36/0	2	Threonine synthase-like 2 (*S. cerevisiae*)	0.000	rs35051888
*LBP*	220/10	20	Lipopolysaccharide binding protein	0.000	rs5744204
	123/6				rs2232582
	238/10				rs2232596
*FGFR4*	267/27	5	Fibroblast growth factor receptor 4	0.000	rs1966265
*SLC44A5*	57/0	1	Solute carrier family 44, member 5	0.000	rs17096508
	40/0				rs10493565
	31/0				rs588098
	41/0				Novel SNV
*FGFR2*	44/0	10	Fibroblast growth factor receptor 2	0.000	rs1047100
*SYTL5*	48/2	X	Synaptotagmin-like 5	0.000	rs5918476
	30/3				rs4827330

Genes under ASE regulation in HCC1954 but expressed from both alleles in HCC1954BL are shown in bold; genes under ASE regulation in HCC1954 but barely expressed in HCC1954BL are shown in regular font. ASE, allele-specific expression; SNV, single nucleotide variant.

A similar data mining process was performed for HCC1954BL. There were 7,848 SNVs in 4,441 genes identified by exome sequencing. Of these, 766 (9.7%) are novel. A total of 3,086 of the 7,848 SNVs were found in 1,918 genes, each of which was represented by at least 20 transcriptome reads. Comparison of SNVs in the exome and transcriptome data suggests that 50 genes are under ASE regulation as demonstrated by 117 SNVs (Table S6 in Additional file 1). The chromosomal distribution of the 1,918 candidate genes and the 50 ASE genes is shown in Figure 3b.

Biological categorization of the 86 ASE genes in HCC1954 shows that many of them are associated with cell-cell signaling and interactions, with 16 encoding cell surface proteins and five encoding extracellular matrix proteins. Of the 16 cell surface proteins, seven are transmembrane receptors, including kinases in the FGFR family and G-protein coupled receptors (Table 2).

For HCC1954 and HCC1954BL combined, approximately two-thirds of the ASE genes had a single SNV locus as supported by the exome data in their CDSs while the remainder had multiple exomic SNVs for ASE concordance (Table 3). In the latter cases, the most significant *P-*value of the ASE locus was used.

Twenty-two ASE genes are shared by both cell lines, and five of these are located on chromosome X. For all shared ASE genes that are not on the X chromosome, the same allele was preferentially expressed in both cell lines, suggesting that common genomic sequence variants are the controlling factors for these ASE events. For 24 genes that display ASE in HCC1954, there was no preferential expression from either allele in HCC1954BL. For 26 ASE genes in HCC1954, it was not possible to determine their status in HCC1954BL because of low or undetectable expression. The remaining 14 ASE genes in HCC1954 have no genotyping status in HCC1954BL due to low exome sequencing coverage, but are likely to be ASE genes in HCC1954BL since 93% of the exome genotypes are in dbSNP, and all have biased allele expression patterns detected in the transcriptome. Only three ASE genes are unique to HCC1954BL, which are expressed in both alleles in HCC1954.

As expected, chromosome X carries ASE genes most frequently in both cell lines. The other ASE genes are distributed across most of the autosomes (Figure 3). Clustering of ASE genes is not observed in the same genomic regions; thus, ASE events are more likely to be individually controlled. Chromosome X harbors none of the unique ASE genes in HCC1954, but two unique ASE genes in HCC1954BL, suggesting that there has been differential escape from X-inactivation between the two cell lines.

Genotyping by exome sequencing and allelotyping by transcriptome sequencing revealed additional genomic aberrations. For example, local genomic disruption at a locus may result in detection of a single allele from transcriptome sequencing. Indeed, in our previous report on transcriptome studies of the same HCC1954 cell line [29], we identified a genomic inversion event at the PHF20L1 gene locus. It was predicted that transcription of PHF20L1 would be impaired for the rearranged allele, leaving the other allele intact. Identification of PHF20L1 as a gene expressed from only one allele in this study agrees with our previous findings. This indirectly demonstrates that our strategy can detect a spectrum of ASE events in the genome.

We identified two additional genes in HCC1954, *GPR56 *and *FAAH2*, for which the transcriptome sequence data were ambiguous. Although each gene is heterozygous at two known SNP loci, only one SNP locus has monoallelic expression while the other distant SNP is expressed from both alleles. We speculate that either local genomic rearrangement or transcription from the opposite strand occurs in HCC1954. It is also possible that there are alternative transcript forms for these two genes, and only one form has unbalanced expression.

### Experimental validation of allele-specific expression events

**Figure 4:**
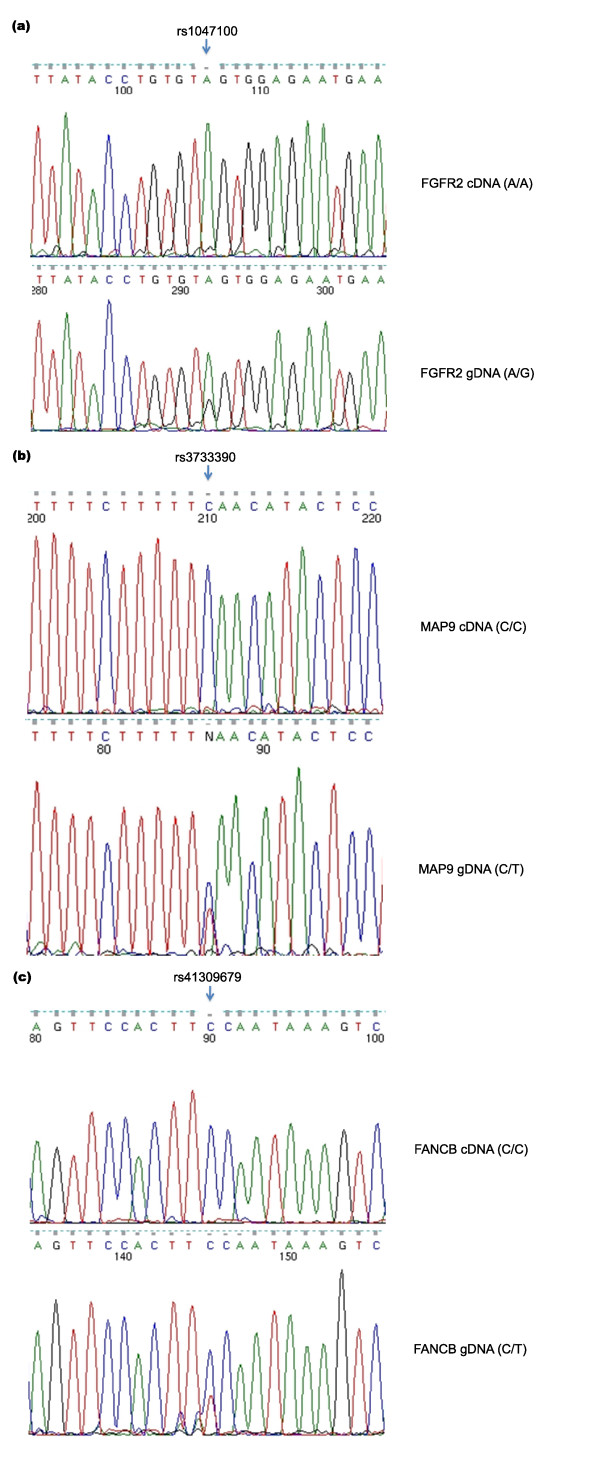
**Validation of allele-specific expression events in HCC1954**. The top trace is from cDNA, and the bottom trace is from genomic DNA (gDNA). **(a) ***FGFR2*; **(b) ***MAP9*; **(c) ***FANCB*.

Three loci were genotyped and allelotyped in HCC1954 to validate three corresponding genes with putative ASE (*FGFR2*, *MAP9*, *FANCB*). PCR was performed to amplify genomic sequences surrounding the SNV loci, while reverse transcription PCR (RT-PCR) was applied to determine if a single allele is preferentially transcribed. Sanger sequencing chemistry was used to confirm the allelic status in both preparations. All three loci were validated as ASE events (Figure 4).

Interestingly, *FGFR2*, a kinase receptor gene, undergoes ASE in HCC1954 (Figure 4a). The *FGFR2 *gene is known to be expressed in multiple alternative splicing forms. It is transcribed in the form of *FGFR2b *in mammary epithelial cells, and *FGFR2c *in surrounding mesenchymal cells [30]. After *de novo *assembly of the Illumina cDNA reads, *FGFR2b *was found to be the only isoform expressed in HCC1954. *FGFR2 *is heterozygous as shown by exonic SNV of rs1047100, which is a synonymous SNV at V232 (GTA versus GTG), but transcribed as *FGFR2b *only from one strand (GTA) as revealed by mRNA reads at rs1047100 (Figure 4a).

Another validated ASE gene is *MAP9 *on chromosome 4, a microtubule-associated protein required for spindle function, mitotic progression, and cytokinesis (Figure 4b). *FANCB*, a member of Fanconi anemia complementation group (FANC) on chromosome X, was also confirmed to be inactivated on one allele in HCC1954 (Figure 4c). Unequal peak heights between two genomic DNA alleles likely result from the pseudo-tetroploidy genome status and copy number variation in HCC1954.

## Discussion

Exome and transcriptome sequencing captures a snapshot of the active genome in a cell population. In addition to revealing SNVs and relative gene expression levels in a sample, the combined data can be used to distinguish active from inactive alleles. By mining sequence data from exomes and transcriptomes, we have identified LOH events and ASE genes in the breast cancer cell line HCC1954 and a lymphoblast cell line from the same individual, HCC1954BL. Our approach demonstrates that the search for genome-wide allele-specific events is feasible with systematic application of sequencing technologies.

Due to its pseudo-tetraploid genomic status with frequent copy number variation in HCC1954, similar numbers of sequence reads often gave lower average coverage of the minor allele in the HCC1954 exome compared to that of the HCC1954BL. Thus, a lower number of high-confidence SNVs detected in HCC1954 is expected. This number would be expected to increase with even greater sequence coverage. After combining with transcriptome sequence data, the SNVs with a minimum of 20× coverage by transcriptome reads were used for ASE mining. We also observed greater variation in mRNA expression levels in the cancer cell line, yielding fewer SNVs with deep transcript coverage for ASE mining. The combined use of exome-capture and transcriptome sequencing focuses on SNVs in genes and captures novel SNVs that were absent from previously published array-based approaches [17,19,31,32].

In general, the total number of heterozygous SNVs detected by the exome-capture sequencing is less than that identified by transcriptome sequencing. This can be attributed to heterozygous allelic variations residing in 5' and 3' UTRs of mRNAs that are not targeted by probes on the exome array. Expansion of targeted regions of the exome array to non-CDS exons would provide additional informative SNVs.

In recent years, experimental evidence has shown that haploinsufficiency of tumor suppressor genes can serve to drive the tumorigenic process [10]. Genetic, epigenetic and environmental factors can modify this haploinsufficiency to promote the tumor phenotype. First, association between LOH and tumor susceptibility is significant only when several tumor suppressor genes are involved in the LOH events [10,33]. Second, in addition to common tumor suppressor genes shared by many cancer types like RB1 and TP53, many tumor suppressor genes are specific to a particular tumor type and/or cell type that originate the tumor. Deficiency of *BRCA1 *and *BRCA2 *is mainly found in breast and ovarian cancers thus far. Third, epigenetic silencing of tumor suppressor genes is achieved by different mechanisms, such as DNA methylation and histone modification. These observations suggest that additional tumor suppressor genes remain to be discovered for specific tumor types.

Many genes identified in our study are either known tumor suppressor genes (for example, *BRCA1*) or previously identified putative tumor suppressor genes (for example, *BCR*). Moreover, genomic instability or epigenetic alterations have been reported in breast cancer and other cancer types for several of the genes in our list. A selection of the LOH and ASE genes and their associated molecular functions is listed in Table 4. For example, LOH is a frequent event for *BRCA1 *in breast and ovarian cancers, for *MSH3 *in breast, bladder and non-small cell lung cancers, and for *PDGFRL *in sporadic hepatocellular carcinomas, colorectal and non-small cell lung cancers. In addition, *FHOD3 *and *MAP2K4 *were previously defined as candidate cancer genes (*CAN *gene) by integrated analysis of homozygous deletions and sequence alterations in breast and colorectal cancers [34]. Meanwhile, epigenetic silencing caused by methylation was previously observed for at least five ASE genes identified in our study, including *DSC3*, *FGFR2 *and *MGMT *in breast cancer and/or in other cancer types. However, a survey of related literature indicates that allelic-specific methylation has not yet been reported for ASE genes identified in this study.

**Table 4:** Selected list of LOH or ASE genes: known or putative tumor suppressor genes

	Gene product and functional properties	Reported functional studies in cancer
LOH genes		
*BRCA1*	Breast cancer 1, a nuclear phosphoprotein involved in maintaining DNA stability	Tumor suppressor function [43]
*MSH3*	MutS homolog 3, a subunit of MutS beta involved in DNA mismatch repair	Genetic instability caused by loss of *MSH3 *in cancers [44]
*PCGF2*	Polycomb group ring finger 2, involved in protein-protein interaction and transcription repression	Tumor suppressor function [45]
*PDGFRL*	Platelet-derived growth factor receptor-like, a cell surface tyrosine kinase receptor	Mutation and gene loss correlated with breast cancer progression [46] and prostate cancer [47]
*BCR*	Breakpoint cluster region	Putative tumor suppressor in meningiomas [48]
		
ASE genes		
*DSC3*	Desmocollin 3, a cell adhesion molecule in cadherin family	Epigenetic silencing of *DSC3 *is a common event in breast cancer [49]
*FGFR2*	Fibroblast growth factor receptor 2, a transmembrane tyrosine kinase	Hypermethylation of *FGFR2 *found in gastric cancer [50]
*MYEOV*	Myeloma overexpressed, a putative transforming gene	Epigenetically inactivated in esophageal squamous cell carcinomas [51]
*TNFRSF10D*	Tumor necrosis factor receptor superfamily, member 10 d, a member of TNF-receptor superfamily	Aberrant methylation in multiple tumor type and mapped to tumor suppressor region in prostate cancer [52,53]
*MGMT*	O-6-methylguanine-DNA methyltransferase, a DNA repair gene	Methylation of MGMT in many types of cancers [41,42,54] and associated with poorer overall and disease-free survival [55]

LOH, loss of heterozygosity; ASE, allele-specific expression.

*FGFR*s, which have been implicated in breast cancer development, are reported to be allele-specifically expressed for the first time in a breast cancer cell in this study. *FGFR2 *has been identified as a risk factor in breast cancer by association studies [30,35-37]. Two intronic SNVs in *FGFR2 *have been reported to increase susceptibility to breast cancer by regulating the downstream gene expression level [35]. *FGFR2 *was identified as a *CAN *gene by combined genomic studies in breast and colorectal cancers [34]. Moreover, prostate and bladder cancers with reduced *FGFR2b *expression show poorer prognosis due to increased potential for invasion and metastasis [38,39]. We can speculate that *FGFR2 *functions as a tumor suppressor in breast cancer, as well as *FGFR4*, for which functions are still unknown.

**Figure 5:**
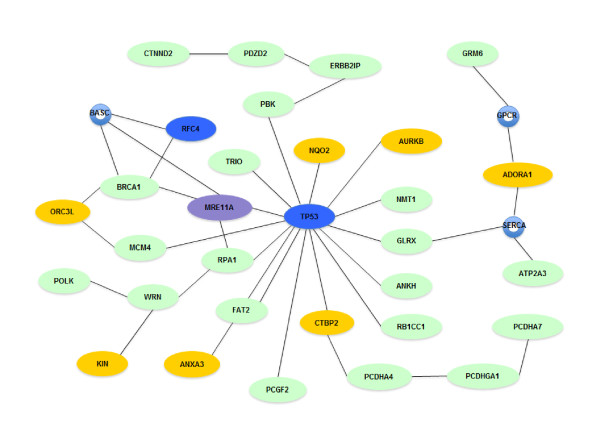
**DNA recombination, replication and repair network**. In HCC1954, genes that have somatic point mutations (blue), ASE (yellow), LOH (green), or translocations (purple) form a DNA repair network. Small circles represent protein complexes or protein families with components encoded by either ASE or LOH genes.

*MGMT *encodes a DNA methyltransferase, a DNA repair protein. The promoter of the *MGMT *gene has been found to be hypermethylated at a high frequency in many types of cancers, including colorectal cancer and glioblastoma [40-42]. This indicates that MGMT may serve as a tumor suppressor in many types of cancer. A protein-protein interaction analysis that integrates all genes that have been found to carry a somatic change in HCC1954, including the LOH and ASE genes identified in this study, genes that carry somatic point mutations [6], as well as a gene mutated by chromosomal translocation [29] yielded a prominent functional network that focuses on DNA recombination, replication and repair (Figure 5). The network is formed by at least 31 molecules composed of 21 genes with LOH, seven genes with ASE, two genes with somatic point mutation and one gene with a translocation.

## Conclusions

Our analysis of the combined effect of LOH and ASE in HCC1954 reveals additional genes that may have tumor suppressor or other functions within this breast cancer cell (summarized in Additional file 1). Recently, several studies have demonstrated the importance of comprehensive characterization of diverse molecular events toward discerning genes and pathways that potentially play a role in tumorigenesis. For example, gene activation can result from various events, such as point mutations that activate a protein product, gene amplification, and gene fusion, as well as epigenetic alteration. Here we demonstrate that the combined approach of exome sequencing and transcript analysis can reveal LOH and ASE events that can each result in haploinsufficiency for specific genes. ASE reflects various types of fluidic genomic alterations, including those that are epigenetic, and thus provides a unique insight to the changing status of cancer cells. This approach will further facilitate the process of identifying additional *CAN *genes and better define drivers of the tumorigenesis process. We note that genetic alterations in immortalized cell lines may not accurately reflect those changes in the cells from which they were derived. Nevertheless, the proof of principle study described here demonstrates that application of this approach to clinical samples such as tumor cells, stromal cells, fibroblasts, and infiltrating T-cells would likely provide additional definition to the significance of ASE in cancer. Our study demonstrates the feasibility of such approaches based on the ever-increasing power of next generation sequencing.

## Materials and methods

### Exome sequencing and mapping

The cell lines HCC1954 and HCC1954 were obtained from ATCC. They were maintained in RPMI medium containing 10% fetal bovine serum, 2 mM L-glutamine and non-essential amino acids. Total DNA was isolated from the cell pellets using the DNeasy Blood and Tissue Kit (Qiagen, Valencia, CA, USA). Genomic DNA was treated with RNase Cocktail™ (Ambion, Austin, TX, USA), followed by phenol-chloroform extraction and precipitation of the aqueous phase in 1/10 volume 3 M sodium acetate and 100% ethanol.

Exome capture was performed using 5 μg of input DNA according to the manufacturer's protocol (Roche Nimblegen, Madison, WI, USA). Briefly, genomic DNA was nebulized for 1 minute using 45 psi of pressure. Sheared DNA fragments were subsequently cleaned with the DNA Clean and Concentrator-25 Kit (Zymo Research, Orange, CA, USA) and a fragment size distribution ranging from 300 to 500 bp was verified via Bioanalyzer (Agilent, Santa Clara, CA, USA). After end-polishing of the genomic fragments, the GS FLX Titanium adaptors were ligated to the sheared genomic fragments. Ligated fragments were next hybridized to the Nimblegen Sequence Capture 2.1 M exome array within Maui hybridization stations, followed by washing and elution of array-bound fragments from the arrays within elution chambers (Nimblegen). Captured fragments were next subjected to 27 rounds of PCR amplification using primers targeting the Nimblegen linkers. Following elution, the capture efficiency was evaluated via quantitative PCR reactions. Six full runs of 454 Titanium were performed for the captured fragments for each cell line. 454 reads were aligned to the human reference genome (hg18) using gsMapper. All raw reads have been deposited to the EBI Sequence Read Archive (SRA; submission ID ERA010917).

### Transcriptome sequencing and mapping

Total RNA was isolated from the cell pellets using the RNeasy Mini Kit (Qiagen). Total RNA was treated with DNase I (New England Biolabs, Ipswich, MA, USA) and purified with Qiagen RNeasy columns (Qiagen). DNA-free RNA yield and purity were initially assessed by spectrophotometry. PolyA+ RNA was prepared from 500 μg of total RNA with oligo(dT) beads using the Oligotex mRNA Mini Kit (Qiagen). First-strand cDNA was prepared from 1 μg of poly(A)+ RNA with 200 pmol oligo random primers by using 300 units of Superscript II reverse transcriptase (Invitrogen, Carlsbad, CA, USA). Second-strand synthesis was performed in 20 μl at 16°C for 2 h after addition of 10 units of *Escherichia coli *DNA ligase, 40 units of *E. coli *DNA polymerase, and 2 units of RNase H (all from Invitrogen). T4 DNA polymerase (5 units) was added and incubated for 5 minutes at 16°C. Double-strand cDNA was purified by phenol-chloroform extraction and precipitation of the aqueous phase in 1/10 volume 3 M sodium acetate and 100% ethanol.

The Illumina GAII sequencing procedure was carried out for paired-end short read sequencing. The RefSeq gene set was queried from NCBI website on 3 December 2009. The approximately 75-Mb dataset comprises 41,249 transcript entries. The longest alternative form for each gene was used as reference in the assembly process. Human reference genome build 36 was also used as the assembly reference. Solexa short reads were mapped to the references using the CLC Bio Genomic Work Bench suite (CLC Bio,8200 Arhus N, Denmark). A stringent cutoff was used, requiring unique read mapping and allowing 2-bp mismatch for each read. Expression level was calculated by RPKM as the number of reads that map per kilobase of exon model per million mapped reads for each gene. Transcriptome reads are accessible through the EBI-SRA (submission ID ERA011762).

### Single nucleotide variant calling

For exome sequencing, SNVs for variant alleles were drawn from the default high-confidence SNV calls by gsMapper (the 454HCDiffs.txt file), which is defined as the variant allele supported by at least three non-duplicated high quality reads with at least 10% variant allele frequency. An annotated SNP file (snp130) was downloaded from NCBI to identify known SNPs. Only SNVs in the CDS were used for downstream LOH and ASE analysis.

For transcriptome sequencing, SNVs were called on all assembled contigs using CLCBio SNV detection tools. A minimum quality of 30 was required for the central SNV base and 15 required for the surrounding bases. A SNV for a minor allele required at least four reads or at least 30% variant allele frequency.

### Statistical significance (*P*-value) of LOH and ASE

Binomial function was used for both LOH and ASE significance to calculate the probability of the reads being randomly distributed between the two alleles.

The cumulative binomial distribution is:

$$B(x;n,p)=\sum_{y=0}^{x}b(y;n,p)$$

For biased reads coverage: *P *= BINOMDIST(#reads for rare allele, #total reads, p_s, TRUE). p_s is probability of success in each trial. For a normal diploid genome like HCC1954BL, 0.5 is applied to p_s. However, the p_s value is adjusted for the HCC1954 genome based on variant allele frequency data from exome sequencing. Multiple correction was also applied to the *P*-values at uneven allelic loci in transcriptome sequencing. We used 2,534 SNVs that have the minimum 20× coverage in the transcriptome for multiple correction calculation in HCC1954.

### Validation of allele-specific expression

In general, genomic DNA flanking the SNV loci to be tested was amplified by using intronic primer pairs. The cDNA fragments were produced by RT-PCR using exonic primer pairs crossing adjacent exons. Sanger sequencing was applied to the amplified genomic DNA and cDNA. Total RNA and DNA from the cell pellets were isolated using the RNeasy Mini Kit and DNeasy Blood and Tissue Kit (Qiagen). Genomic DNA was treated with RNase Cocktail™ (Ambion), followed by phenol-chloroform extraction and precipitation of the aqueous phase in 1/10 volume 3 M sodium acetate and 100% ethanol. Total RNA was treated with DNase I (New England Biolabs) and purified with Qiagen RNeasy columns (Qiagen). DNA-free RNA yield and purity were assessed by spectrophotometry and denaturing agarose gels. A total of 0.5 to 1.0 μg of RNA was reverse transcribed into cDNA by using an Omniscript RT kit according to the manufacturer's protocol using oligo (dT)_18 _primers. PCRs from the genomic DNA and RT-PCR were undertaken using High-Fidelity Platinum Taq (Invitrogen) plus 10 pmol of each of the primers listed in Table S7 in Additional file 1. After gel purification, the amplicons were submitted to Sanger sequencing with the PCR primers.

### Molecular functional network

Primary molecular functions and networks involved were analyzed with the IPA software developed by Ingenuity (Redwood City, CA, USA).

## Abbreviations

ASE: allele-specific expression; BP: base pair; CDS: coding sequence; LOH: loss of heterozygosity; RT-PCR: reverse transcription PCR; SNP: single nucleotide polymorphism; SNV: single nucleotide variant; SRA: Sequence Read Archive; UTR: untranslated region.

## Authors' contributions

QZ, EFK and PAG performed data analysis; OLC and RBP performed the experiments; LE, SK and ZY generated the data; QZ, EFK, SL, ARV, BR, SJdS, AAC, AJGS and RLS contributed to research design and discussion of the manuscript; QZ, ERK and RLS wrote the manuscript. All authors read and approved the final manuscript.

## Supplementary Material

Additional file 1 **Supplemental tables**. Table S1: exome genotyping in HCC1954. Table S2: exome genotyping in HCC1954BL. Table S3: LOH genes in HCC1954. Table S4: LOH genes in HCC1954BL. Table S5: ASE genes in HCC1954. Table S6: ASE genes in HCC1954BL. Table S7: primers used in ASE events validation. Table S8: summary of putative tumor suppressor genes identified by LOH and ASE events. Click here for file

Additional file 2 **Supplemental figures**. LOH events detected across each chromosome. Click here for file
